# Consistently dated Atlantic sediment cores over the last 40 thousand years

**DOI:** 10.1038/s41597-019-0173-8

**Published:** 2019-09-02

**Authors:** Claire Waelbroeck, Bryan C. Lougheed, Natalia Vazquez Riveiros, Lise Missiaen, Joel Pedro, Trond Dokken, Irka Hajdas, Lukas Wacker, Peter Abbott, Jean-Pascal Dumoulin, François Thil, Frédérique Eynaud, Linda Rossignol, Wiem Fersi, Ana Luiza Albuquerque, Helge Arz, William E. N. Austin, Rosemarie Came, Anders E. Carlson, James A. Collins, Bernard Dennielou, Stéphanie Desprat, Alex Dickson, Mary Elliot, Christa Farmer, Jacques Giraudeau, Julia Gottschalk, Jorijntje Henderiks, Konrad Hughen, Simon Jung, Paul Knutz, Susana Lebreiro, David C. Lund, Jean Lynch-Stieglitz, Bruno Malaizé, Thomas Marchitto, Gema Martínez-Méndez, Gesine Mollenhauer, Filipa Naughton, Silvia Nave, Dirk Nürnberg, Delia Oppo, Victoria Peck, Frank J. C. Peeters, Aurélie Penaud, Rodrigo da Costa Portilho-Ramos, Janne Repschläger, Jenny Roberts, Carsten Rühlemann, Emilia Salgueiro, Maria Fernanda Sanchez Goni, Joachim Schönfeld, Paolo Scussolini, Luke C. Skinner, Charlotte Skonieczny, David Thornalley, Samuel Toucanne, David Van Rooij, Laurence Vidal, Antje H. L. Voelker, Mélanie Wary, Syee Weldeab, Martin Ziegler

**Affiliations:** 1 LSCE/IPSL, Laboratoire CNRS-CEA-UVSQ, 91191 Orme des Merisiers, France; 2 Ifremer, Unité de Geosciences Marines, 29280 Plouzané, France; 3 Uni Research, Nygårdsgaten 112, 5008 Bergen, Norway; 4 Laboratory of Ion Beam Physics, ETH Zürich, 8093 Zürich, Switzerland; 5 School of Earth and Ocean Sciences, Cardiff University, CF10 3AT Cardiff, UK; 6 Institute of Geological Sciences and Oeschger Center for Climate Change Research, University of Bern, 3012 Bern, Switzerland; 7 LMC14, Université Paris-Saclay, F-91191 Gif-sur-Yvette, France; 8 EPOC, Université Bordeaux, Allée Geoffroy St Hilaire, 33615 Pessac, France; 9 LOOP, Universidade Federal Fluminense, Niterói, RJ Brazil; 10 Leibniz-Institute for Baltic Sea Research Warnemünde, Seestrasse 15, 18119 Rostock, Germany; 11 University of St Andrews, St Andrews, Scotland, KY16 9AL UK; 12 University of New Hampshire, 56 College Road, Durham, NH 03824 USA; 13 Oregon State University, Corvallis, OR 97331 USA; 14 GeoForschungsZentrum, 14473 Potsdam, Germany; 15 Ecole Pratique des Hautes Etudes (EPHE, PSL), 4-14 rue Ferrus, 75014 Paris, France; 16 Royal Holloway University of London, Egham, Surrey TW20 0EX UK; 17 LPG-Nantes, Université de Nantes, 44300 Nantes, France; 18 Hofstra University, Hempstead, NY 11549-1140 USA; 19 Lamont-Doherty Earth Observatory, Columbia University, 61 Route 9W - PO Box 1000, Palisades, NY 10964-1000 USA; 20 Uppsala University, Geocentrum, Villavägen 16, SE-752 36 Uppsala, Sweden; 21 Woods Hole Oceanographic Institution, 266 Woods Hole Rd., Woods Hole, MA 02543-1050 USA; 22 University of Edinburgh, School of Geosciences, Edinburgh, EH9 3FE UK; 23 Geological Survey of Denmark and Greenland, Øster Voldgade 10, 1350 Copenhagen, Denmark; 24 IGME - Instituto Geológico y Minero de España, Calle Ríos Rosas, 23, 28003 Madrid, Spain; 25 University of Connecticut, 1080 Shennecossett Road, Groton, CT 06340 USA; 26 Georgia Institute of Technology, 311 Ferst Drive, Atlanta, GA 30332-0340 USA; 27 INSTAAR, University of Colorado, Boulder, CO 80303 USA; 28 AWI, Am Alten Hafen 26, 27568 Bremerhaven, Germany; 29 IPMA-DivGM, Avenida Doutor Alfredo Magalhães Ramalho, 6, 1495-165 Alges, Portugal; 30 CCMAR, Universidade do Algarve, Campus de Gambelas, 8005-139 Faro, Portugal; 31 LNEG, Bairro do Zambujal, 2610-999 Amadora, Portugal; 32 GEOMAR, Wischhofstrasse 1-3, 24148 Kiel, Germany; 33 UK British Antarctic Survey, Madingley Road, Cambridge, CB3 0ET UK; 34 Vrije Universiteit Amsterdam, De Boelelaan 1087, 1081 HV Amsterdam, Netherlands; 35 Université de Bretragne Occidentale, Technopôle Brest-Iroise, 29280 Plouzané, France; 36 MARUM, University of Bremen, D-28359 Bremen, Germany; 37 Max Planck Institute, Hahn- Meitner- Weg 1, 55128 Mainz, Germany; 38 Thermo Fisher Scientific, Hanna-Kunath Straße 11, Bremen, 28199 Germany; 39 Geozentrum Hannover, Stilleweg 2, 30655 Hannover, Germany; 40 University of Cambridge, Godwin Laboratory for Palaeoclimate Research, Cambridge, CB2 3EQ UK; 41 GEOPS, Université de Paris Sud, 91405 Orsay, France; 42 University College London, Gower Street, London, WC1E 6BT UK; 43 Ghent University, Krijgslaan 281, 9000 Gent, Belgium; 44 Aix-Marseille Université, CNRS, IRD, INRA, Coll France, CEREGE, Europole de l’Arbois, 13545 Aix-en-Provence, France; 45 ICTA, Universitat Autònoma de Barcelona, 08193 Bellaterra, Barcelona Spain; 46 University of California Santa Barbara, Santa Barbara, 1006 Webb Hall, CA 93106-9630 USA; 47 University of Utrecht, Princetonlaan 8a, 3584 CB Utrecht, Netherlands

**Keywords:** Palaeoclimate, Palaeoceanography

## Abstract

Rapid changes in ocean circulation and climate have been observed in marine-sediment and ice cores over the last glacial period and deglaciation, highlighting the non-linear character of the climate system and underlining the possibility of rapid climate shifts in response to anthropogenic greenhouse gas forcing. To date, these rapid changes in climate and ocean circulation are still not fully explained. One obstacle hindering progress in our understanding of the interactions between past ocean circulation and climate changes is the difficulty of accurately dating marine cores. Here, we present a set of 92 marine sediment cores from the Atlantic Ocean for which we have established age-depth models that are consistent with the Greenland GICC05 ice core chronology, and computed the associated dating uncertainties, using a new deposition modeling technique. This is the first set of consistently dated marine sediment cores enabling paleoclimate scientists to evaluate leads/lags between circulation and climate changes over vast regions of the Atlantic Ocean. Moreover, this data set is of direct use in paleoclimate modeling studies.

## Abstract (summary)

**Table:** 

Design Type(s)	modeling and simulation objective • data collection and processing objective • source-based data analysis objective
Measurement Type(s)	age-depth model
Technology Type(s)	computational modeling technique
Factor Type(s)	geographic location • depth
Sample Characteristic(s)	Atlantic Ocean • marine sediment

Machine-accessible metadata file describing the reported data (ISA-Tab format)

## Background & Summary

In order to decipher the mechanisms at play in observed past climate changes, it is necessary to establish a common temporal framework for paleoclimate records from different archives and from different locations. Determining the lead/lag relationships between different climatic and circulation changes can help to identify the underlying causes and foster development of conceptual hypotheses to be tested with climate model simulations. Also, paleoclimate data-model integration studies, such as groundtruthing of transient modeling analyses, timeslice comparisons of proxy data, or data assimilation, necessitate consistent paleoclimate records chronologies in calendar years.

Here we focus on the last 40 ky because it is the time span covered by radiocarbon dating and the sole period for which it is possible to establish calendar age timescales for marine cores with a precision approaching that of ice core or speleothem records.

Radiocarbon dating of marine records is complicated, however, by a difference between the surface water ^14^C/^12^C ratio (expressed as ∆^14^C, in ‰) and that of the contemporaneous atmosphere, due to the balance between the input of atmospheric ^14^C and its removal by radioactive decay in the water column, advection, and mixing with older waters. This difference in ∆^14^C is termed the “reservoir age” of the surface waters. Previous studies have revealed that surface reservoir ages have not remained constant over time at high latitudes of the North Atlantic and Southern Ocean (i.e. poleward of ~38°N and of ~40°S) due to changes in the location and vigour of deep-water formation^1–4^.

In those high-latitude regions, it is thus necessary to use an alternative dating strategy in lieu of ^14^C dating of marine organisms. Here we adopt a strategy that has been widely applied (e.g. refs^4–7^) and has been adopted by the INTIMATE (Integration of Ice core, Marine and Terrestrial records of the North Atlantic) group when surface reservoir ages can not be assessed^8^. This strategy consists of synchronizing the sea surface temperature (SST) signal recorded in marine cores with the air temperature signal recorded in polar ice cores. This dating approach is based on the observed thermal equilibrium between the ocean’s surface water and overlying air. Previous studies have demonstrated that changes in air and sea surface temperature were synchronous across the last deglaciation^9^ and some of the last glacial rapid climate changes^10^ over the North Atlantic region. Moreover, modeling studies of the last deglaciation^11^ or last glacial millennial climate changes^12,13^ show that both increases and decreases in North Atlantic (Southern Ocean) SST and in air temperature above Greenland (Antarctica) are synchronous.

Currently, the Greenland NorthGRIP (NGRIP) ice core can be considered the best-dated continuous continental paleoclimatic archive over the last 50 to 75 ky. The NGRIP Greenland Ice Core Chronology 2005 (GICC05) calendar age scale has been established by annual layer counting with estimated uncertainties of 50 y at 11 calendar ky BP (i.e. calendar ky before 1950, noted ka hereafter), 100 to 450 y for the 11–30 ka interval, and 450 to 800 y for 30–40 ka^14^ (y or ky referring to durations and ka to dates). Moreover, a common chronology for Greenland and Antarctica ice cores has been developed based on their records of ^10^Be and atmospheric CH_4_ concentration^15,16^. This dating effort yielded the Antarctic AICC2012 age scale for four Antarctic ice cores, which is fully consistent with the GICC05 age scale over the last 60 ky^16^. Using the GICC05 and AICC2012 age scales as alignment targets for high latitude SST records of the north and south hemispheres respectively, it is thus possible to directly compare marine records from both hemispheres on a common time frame.

**Fig. 1:**
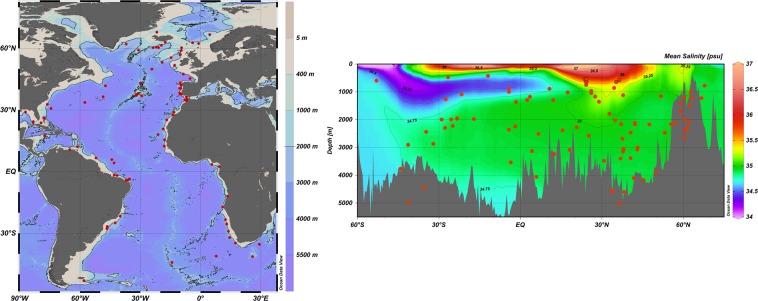
Location of the 92 dated Atlantic sediment cores (see Online-only Table 1 for precise coordinates and water depths of the cores). The figures were generated using the Ocean Data View software^53^, the ETOPO bathymetry^54^ (left panel), and the WOA13 mean annual salinity^55^ along a mid-Atlantic north-south section (right panel). The salinity section illustrates the distribution of the cores with respect to the main modern water masses.

Here, we present the first set of consistently dated Atlantic sediment cores from 92 locations distributed between 68°N and 53°S, and between 400 and 5000 m water depth (Fig. 1, Online-only Table 1, ref.^17^), together with consistently derived dating uncertainties. This new data set enables paleoclimate scientists to (i) examine relative phases between Atlantic records (e.g. planktonic and benthic oxygen and carbon isotopes, Pa/Th); and (ii) use the spatial and temporal changes recorded in Atlantic sediments to constrain paleoclimate model simulations.

## Methods

We compiled existing paleoceanographic data from Atlantic sediment cores covering part of or the entire 0–40 ka interval, with sedimentation rates of at least 5 cm/ky, for which there exists the following dating means: radiocarbon dates for mid and low latitudes sediment cores, and SST or magnetic records for sediment cores located poleward of ~38°N and ~40°S. New cores were added to fill gaps with respect to the available geographical and water depth coverage, and additional radiocarbon dates were produced to improve the existing age models of some cores (Online-only Table 1).

In mid and low latitudes (i.e. between ~40°S and ~38°N), reservoir ages can be assumed not to have strongly varied in response to ocean circulation changes of the last glacial and deglaciation. The same is true at all latitudes during the Holocene. Thus, in mid and low latitudes, and during the Holocene at higher latitudes, the sediment cores were dated by means of calibrated radiocarbon ages. For this, 1427 published and 104 new radiocarbon dates have been calibrated using the Bayesian calibration program “MatCal”^18^, and the IntCal13 and SHCal13 calibration curves^19,20^ for North and South Atlantic cores, respectively.

**Fig. 2:**
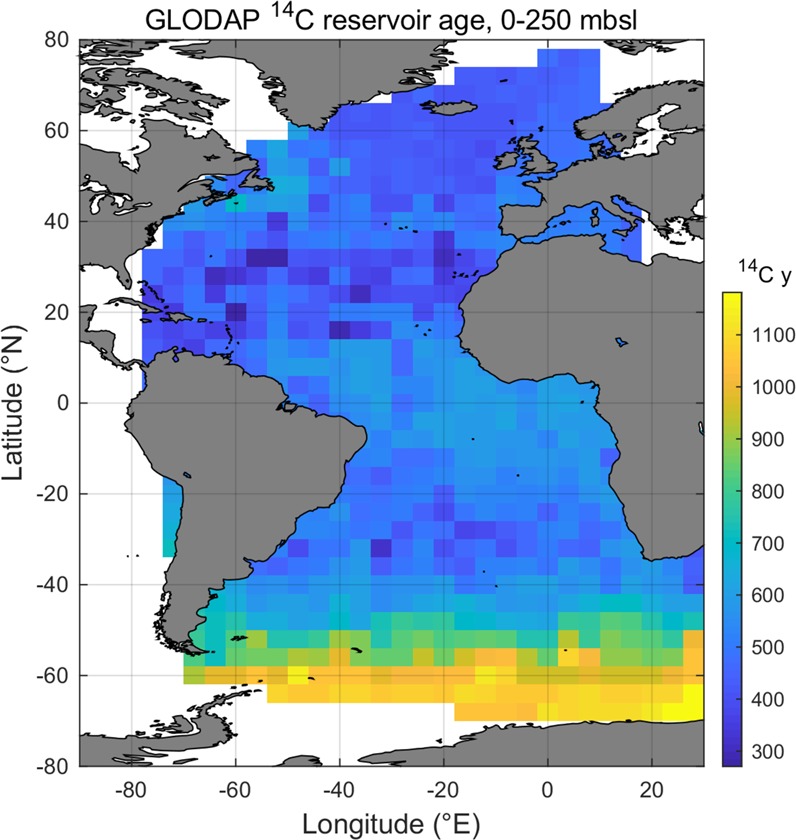
Average reservoir age extracted from the GLODAP data re-gridded to a 4° × 4° grid and averaged over the upper 250 m of the water column. These values can be downloaded from Figshare^56^.

We accounted for both spatial and temporal variability in ^14^C reservoir ages. To estimate spatial variations in reservoir ages we extracted bomb-corrected reservoir ages from the GLobal Ocean Data Analysis Project for Carbon (GLODAP) data set^21^. Prior to extracting these surface reservoir ages, GLODAP data were re-gridded to a 4° × 4° grid, whereby the mean and standard deviation for the GLODAP data points from the upper 250 m for each 4° × 4° grid cell were calculated. The modern surface water reservoir age at a given site is then obtained from the nearest grid node to the core site (Fig. 2). In the case of certain sites that are out of range of the GLODAP grid, such as those in the Gulf of Mexico, we have extrapolated the GLODAP 4°x4° grid to these areas. This spatially varying component of the reservoir age is subtracted from the laboratory ^14^C age before calibration (with error propagation). The error used for this spatial reservoir age component is either the computed GLODAP standard deviation, or 100 ^14^C yr, whichever is greater. For pre-Holocene dates, a minimum of 200 ^14^C yr is used instead of 100 ^14^C yr.

To also consider temporal changes in reservoir age, we further applied a correction to account for the impact of atmospheric CO_2_ concentration changes upon surface water ^14^C activity. At the Last Glacial Maximum (LGM), the lower atmospheric CO_2_ concentration induced an increase in atmospheric ∆^14^C of ~30‰ due to the speciation change, everything else being equal^22^. This ~30‰ increase in atmospheric ∆^14^C in turn caused a ~250 y increase in surface water reservoir ages^22^. To account for this temporal change in surface reservoir age, we linearly scaled a reservoir age correction to atmospheric pCO_2_, whereby a correction of 0 ^14^C y corresponds to present day pCO_2_, and 250 ^14^C y to LGM pCO_2_. For pCO_2_ values, we consulted the composite atmospheric CO_2_ record of Antarctic ice cores^23^. This age-dependent component of the reservoir age is added to the IntCal13 (or SHCal13) ^14^C age record before calibration.

Even in regions where surface reservoir ages can be predicted based on the evolution of atmospheric CO_2_, as described above, increased uncertainties in radiocarbon-dated chronologies can still arise from bioturbation biases (e.g. ref.^24^). Thus, in the best cases, when bioturbation biases and local changes in past surface reservoir ages remain limited, sediment core dating uncertainties mainly arise from the conversion of radiocarbon ages into calendar ages. In these cases, uncertainties are less than 150 y for the time interval 0–11 ka, of about 400 y for the 11–30 ka interval, and of 600 to 1100 y for the 30–40 ka interval^19^. In all other cases, dating uncertainties are larger.

Almost all our age-depth models of low- and mid-latitude cores (51 out of the 92 cores, see Online-only Table 1) are entirely based on calibrated ^14^C ages. In three cores (GeoB3910, MD09-3246 and MD09-3256Q), located on the Brazilian margin in a region under the influence of the Intertropical Convergence Zone, it is possible to take advantage of the simultaneous recording of rainfall increases during Greenland stadial periods in the marine cores and in U-Th dated speleothems from the adjacent continent to improve the marine age models. Rainfall increases are recorded both by XRF-Ti/Ca peaks in the marine cores, and by δ^18^O decreases in the speleothems^25^. By aligning the XRF-Ti/Ca in the marine cores to the speleothem δ^18^O, it is possible to improve the precision of the marine age models around 40 ka and to extend them beyond the limit of ^14^C dating. Importantly, the speleothem record from El Condor cave^26^, to which we have aligned the three marine cores, has been shown to be in phase, within dating uncertainties, with the NGRIP air temperature record in the GICC05 age scale^25,27^. Our alignment of GeoB3910, MD09-3246 and MD09-3256Q marine cores to El Condor speleothem is thus consistent with the NGRIP GICC05 age scale.

For cores located north of ~38°N (26 cores) and south of ~40°S (2 cores), and ODP Site 1060 for which there exist no ^14^C dates but where planktonic foraminifer census counts exhibit a clear NGRIP signal^28^, we have used calibrated radiocarbon ages only over the Holocene portion (i.e. after the end of the Younger Dryas, dated at 11.65 ka in the GICC05 age scale^29^), and aligned their glacial and deglacial portions to NGRIP or EPICA Dronning Maud Land (EDML) air temperature signal. We used different types of chronological markers to derive these 29 age-depth models:

1. Tie points defined by aligning high latitude SST records to NGRIP air temperature proxy record on the GICC05 age scale for North Atlantic cores, and to EDML air temperature on the AICC2012 age scale for South Atlantic cores;
2. Tie points defined by aligning magnetic properties of northern North Atlantic and Nordic Seas cores to the NGRIP air temperature signal on the GICC05 age scale;
3. Dated tephra layers.

The dating procedures (1)-(3) are described in detail below. The alignment procedures (1) and (2) by essence impede the assessment of leads and lags between the aligned records. For instance, leads/lags between SST and polar air temperatures, or among SST records from high latitude marine cores, are by construction not significantly different from zero. In contrast, this dating approach gives access to the relative timing of circulation changes recorded at different water depths in cores located on depth transects.

(1) We aligned SST records to polar ice core air temperature proxy records using the AnalySeries program^30^. NGRIP alignment targets correspond to the rapid transitions out of and into Greenland stadials, as dated and listed in refs^29,31^ (Online-only Table 2). Tie points were generally defined by aligning rapid warmings recognized in both the ice core and marine core, as recommended in ref.^8^. In rare cases, rapid and well-defined coolings have been aligned. In a few cases, when the SST record resolution was too low or the signal shape ambiguous, maxima or minima have been aligned. Remaining ambiguities in the identification of alignment tie points were solved in most cases by fulfilling the condition that the tie point age is younger or equal to the calibrated ^14^C ages obtained by assuming no other change in surface reservoir age than the temporal evolution due to changing atmospheric pCO_2_. Not fulfilling this condition would result in negative surface reservoir ages, which is not physically possible (see Supplementary Fig. 1 for an example).

SST alignment to Antarctic temperature variations was made at marked transitions in the temperature record, such as Antarctic Isotopic Maxima^32^, the onset of the early and late deglacial warming, or the beginning of the Antarctic Cold Reversal.

In addition, we used the following three alignment targets in the North Atlantic:

(i) A first alignment target is based on the observation that the cooling marking the beginning of Heinrich Stadial 1 in three independently dated North Atlantic cores is synchronous with the sharp increase in dust flux recorded in the Greenland ice cores and dated at 17.48 ka ± 0.21 ky on the GICC05 age scale^33^. This observation is consistent with this cooling being coeval with an increase in dust transport from Asia to Greenland, as observed during other Greenland stadials^34^.
(ii) Two other alignment targets correspond to the beginning and the end of the warm event identified in ref.^35^ within Greenland stadial 3 (GS-3) in several North Atlantic cores between 24 and 25 ka. This warm event within GS-3 is not clearly recorded in Greenland ice (δ^18^O) or gas (δ^15^N) isotopic records, but corresponds to a marked decrease in dust flux. Here again, we aligned the beginning and end of the warm event to the corresponding changes in the NGRIP dust flux dated on the GICC05 age scale at 25.05 ka ± 0.35 ky and 24.1 ka ± 0.33 ky, respectively.

For consistency, the alignment tie points in high latitude cores were all defined by the same person. Similarly, one single person defined all the alignment tie points in the three Brazilian cores. Also, the SST records used in the present study are all based on planktonic foraminifer census count data. When SST reconstructions based on full census count data were not available, we used the percentage of the polar species *Neogloboquadrina pachyderma* (left coiling) as a proxy for SST. This approach has been described and validated in a number of studies (e.g. refs^36–39^). In two North Atlantic cores (ODP Site 1060 and core MD08-3180Q) we used the percentage of warm species instead, because the percentage of *N*. *pachyderma* was too low. In the particular case of the Iberian margin, it has been shown that *Globigerina bulloides* δ^18^O co-varies with SST^40,41^ and we have used *G*. *bulloides* δ^18^O as a proxy for SST when no SST estimates were available.

Both age and depth uncertainties are defined for each tie point. The depth uncertainty directly depends on the sampling resolution of the SST curve: it is taken as half of the depth interval corresponding to the rapid warming (or more rarely cooling), or as half of the width of the SST maximum or minimum, when maxima or minima have been aligned. In instances of ambiguities that could not be tested by the constraints provided by ^14^C dates, we attributed an uncertainty to the depth of the tie point, large enough to encompass the two events (warmings, or more rarely, coolings or SST maxima or minima) which could both be aligned to the same target. The uncertainty on the tie point ages is the GICC05 dating precision of the transitions between Greenland stadials and interstadials, with one sigma uncertainties defined as half the cumulative ‘maximum counting error’ in the GICC05 age scale^29,31^. Similarly, the dating uncertainty of the alignment tie points defined with respect to AICC2012 is the dating error given in ref.^16^.

(2) In high northern latitudes, when SST records are not available, for some cores it is possible to instead use high-frequency variations in magnetic susceptibility (MS) recorded during the last glacial period. The rapid oscillations in magnetic properties in sediment cores on the flow path of North Atlantic Deep Water (NADW) in the Nordic Seas and North Atlantic have indeed been shown to be in phase with the Greenland ice δ^18^O or air temperature signal^42^. Support for this synchronicity comes from tephra and geomagnetic field (Laschamp inclination excursion) marine records. These marine records become aligned with tephra and cosmogenic nuclide Greenland records when the MS tuning to Greenland is applied (e.g. refs^43–45^).

We dated five cores located north of 62°N by aligning their MS records to the NGRIP ice δ^18^O signal (Online-only Table 1). MS tie points and their associated uncertainties were defined using the same method as described for the alignment of SST signals to ice core records. The MS records of four of these five cores have been previously shown to be in phase with the Greenland air temperature signal^42^. More recently, the identification of tephra layers in core MD99-2284 demonstrated that this core’s MS record is also in phase with the NGRIP δ^18^O record^43^. This can be explained by the fact that changes in MS arise from changes in the efficiency of the transport of fine grained magnetic particles by deep currents from the source to the site of deposition^42^. The fact that the MS signal is in phase in cores located north and south of the sills separating the Nordic Seas from the North Atlantic basin, suggests that the source of magnetic minerals could be at the sills, with the strength of the overflow from the Nordic Seas directly proportional to the strength of the inflow into the Nordic Seas.

(3) We used dated tephra layers as additional chronological markers over the last 55 ky in 10 of the northernmost cores (Online-only Table 1). The following four tephra layers have been recognized both in Greenland ice cores and in certain North Atlantic and Nordic Seas marine cores: the Saksunarvatn Ash^46^, the Vedde Ash^46^, the Faroe Marine Ash Zone (FMAZ) II^46,47^, and the widespread rhyolitic component of North Atlantic Ash Zone (NAAZ) II (II-RHY-1)^46,48^ (Online-only Table 2).

**Fig. 3:**
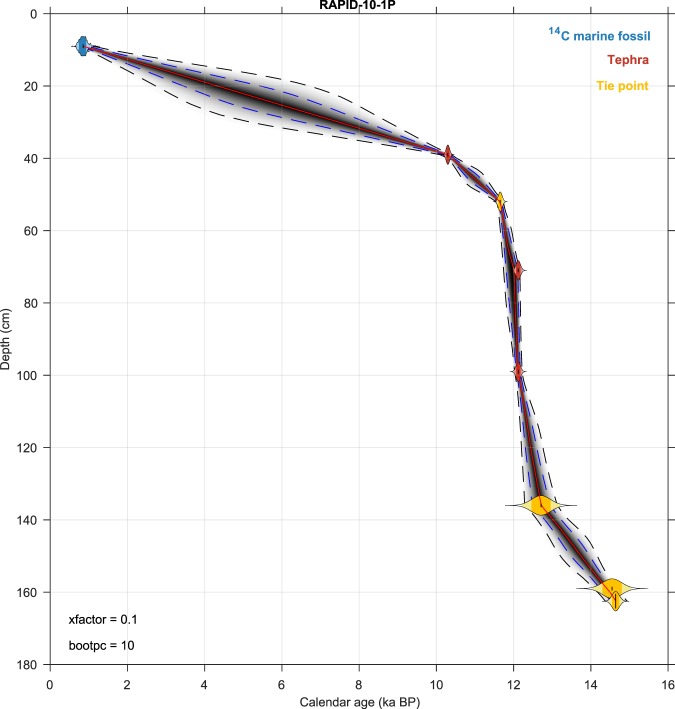
Example of age-depth plot produced by *Undatable*. Age-depth model produced for North Atlantic core RAPID-10-1P with bootstrapping set to 10% and sedimentation rate uncertainty set to 0.1 (see ref.^49^ for details). Blue, yellow and red probability density functions indicate the radiocarbon and alignment tie points, and tephra age-depth constraints, respectively. The grey cloud indicates the probability density cloud of the age-depth model, whereby darker colors indicate higher age-depth probability. The blue and black broken lines represent 68.27% and 95.45% confidence intervals, respectively. The red line indicates the age-depth model median.

Age-depth relationships were built for each core accounting for both the age and depth uncertainties of the ^14^C dates and chronological markers, using the age-depth modeling routine “*Undatable*”^49^ (Fig. 3). This new rapid age-depth modeling routine was ideal for this project as it allowed us to run and re-run age models for the many sediment cores that we have analyzed. Moreover, this age-depth modeling routine computes a conservative age-depth uncertainty, through the provision of bootstrapping and sediment accumulation rate uncertainty^49^ (Fig. 3). Default values for bootstrapping percentage and sedimentation rate uncertainty were set to 10% and 0.1 respectively. In the presence of age reversals, we progressively increased the bootstrapping percentage in order to make sure that the dating uncertainty computed by *Undatable* was large enough to encompass most calibrated ^14^C ages, leaving out only outliers beyond 2 sigma dating uncertainty. This way, we take into account increased dating uncertainty associated with the existence of age-depth scatter, which may be related to sedimentation hiatuses, abundance changes, or bioturbation. Also, we considered tephra layers as the most reliable age-depth constraints and, therefore, *a priori* excluded them from the bootstrapping process (e.g. ref.^50^).

In some North Atlantic cores (7 out of 92, cf. Online-only Table 1), we used ^14^C dates together with SST alignment tie points to NGRIP. These cores are located at the northern edge of the region where surface reservoir ages may be assumed not to have strongly varied in response to ocean circulation changes, and are characterized by large changes in SST which parallel the NGRIP ice δ^18^O signal. In those cores, we used alignment tie points to complement calibrated ^14^C dates when the latter were too sparse.

Finally, although the focus of this work is the time interval 0–40 ka, we used dating information available beyond 40 ka to ensure the robustness of the computed sedimentation rate and age-depth relationship around 40 ka.

## Data Records

The present set of age-depth models contains three text files per marine sediment core^17^. The first text file (“age depth input”) contains an overview of the ^14^C ages and other age constraints used in the age-depth model. More specifically, a first section provides all the available ^14^C raw data, the reservoir age and calibration curve used, as well as the calibrated ages together with the 68.3% highest posterior density interval(s), and specifies which ^14^C dates have been used to generate the age-depth model. A second section provides the definition of the alignment tie points: the tie points depth and its uncertainty, the tie points age and its uncertainty, the nature of the tie points and the nature of the uncertainty of the tie points age. The second text file (“udinput”) contains the input for the age-depth model in the *Undatable* format. The third text file (“_admodel_ka”) contains the computed age-depth relationship and associated dating uncertainties. In addition to the complete set of data records archived on Seanoe^17^, the 92 “_admodel_ka” text files can be found on Pangaea^51^.

Notably, the fact that the ^14^C raw data are provided makes the present data set easy to update using a future ^14^C calibration curve. Also, tie point depths are provided, allowing updates of the age-depth models if higher resolution SST records are produced.

**Fig. 4:**
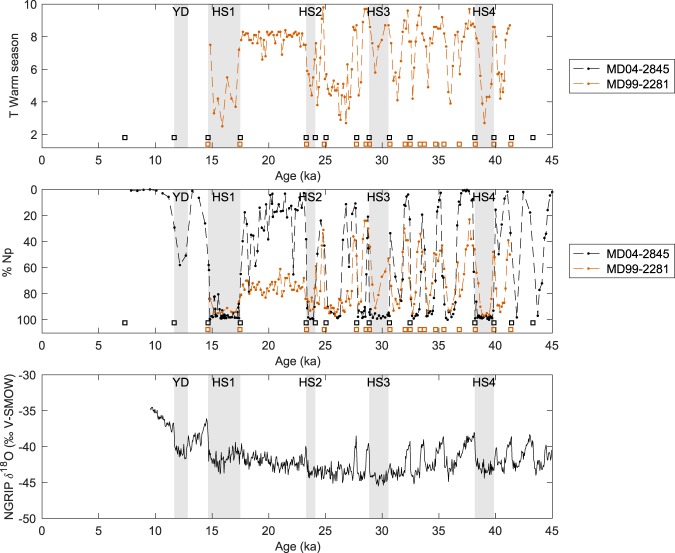
Example of North Atlantic and Nordic Seas cores dated by alignment of their SST records to the NGRIP ice δ^18^O signal. Top panel: planktic foraminifer-based warm season surface temperature of core MD99-2281^57,58^; middle panel: % *N*. *pachyderma* of core MD99-2281 and MD04-2845^59,60^ (both panels: diamonds and squares above the x-axis indicate calibrated ^14^C ages and alignment tie points, respectively). Bottom panel: NGRIP ice δ^18^O record on the GICC05 age scale^61^. Grey bands highlight the Younger Dryas and Heinrich stadials 1–4 chronozones as defined in Online-only Table 2.

In addition to these three text files, the age-depth model plot produced by the *Undatable* routine (see Fig. 3 for an example) is provided for each core, as well as a plot of the aligned SST or MS record, ice core record, and chosen alignment tie points (see Fig. 4 for an example) for the cores which have been partially or completely dated by alignment to an ice core record^17^.

## Technical Validation

The information relative to the validation of the age models entirely based on ^14^C dates can be found in the publications describing the *Undatable* age-depth modeling routine^49^ and the “MatCal” Bayesian calibration program^18^. The age-depth model plot provided for each core (e.g. Fig. 3) shows the calibrated ^14^C dates together with the computed age-depth relationship and dating uncertainty, as well as the bootstrapping percentage and sedimentation rate uncertainty values used in the computation.

Concerning the age models based on the alignment of SST to NGRIP air temperature, a first validation step involved comparing the resulting dated SST signals of different marine cores among themselves and with the NGRIP air temperature signal. An illustration of such a comparison is given in Fig. 4 for North Atlantic core MD04-2845 and Norwegian Sea core MD99-2881. Moreover, available ^14^C data over glacial and deglacial portions of cores dated by alignment to NGRIP provide a verification of the tie points selection since surface water reservoir ages should not be negative (e.g. Supplementary Fig. 1). Interestingly, around Heinrich stadial 4 (38 to 40 ka), our age-depth models yield ages which are systematically older than the calibrated ages obtained using IntCal13 and modern surface water reservoir age values, in agreement with the recent findings of ref.^52^ showing that IntCal13 is too young with respect to GICC05 during that time interval.

The age models based on the alignment of MS to NGRIP ice δ^18^O have been validated by comparing the resulting dated MS signals with the NGRIP ice δ^18^O signal (Supplementary Fig. 2). Moreover, these age models have been validated by climate-independent tie points, such as tephra layers in core MD99-2284^43^, MD95-2010^44^ and ENAM93-21^45^, or changes in the Earth’s magnetic field intensity in core MD99-2281^44^.

The age models making use of the alignment of Ti/Ca to speleothem isotopic records have been validated by comparing the radiocarbon-dated upper portion of the cores with the U-Th dated speleothem signal (Supplementary Fig. 3). This validation was the initial step that led to the use of speleothem isotopic records to complement the dating of the three cores from the Brazilian margin since it demonstrates that terrigenous input at these sites is coeval with the precipitation events recorded in the speleothems^25^.

## Supplementary Information

### ISA-Tab metadata file

Download metadata file

### Supplementary information

Supplementary figures

## Data Availability

The *Undatable* software was used to create age-depth models based on the age-depth constraints given in the “udinput” text files for each core. The software and accompanying source code can be downloaded from the *Zenodo* public archive (10.5281/zenodo.2527642).

## Online-only Tables

**Online-only Table 1:** Dated cores and dating methods used outside of the Holocene.

Core name	Latitude, decimal degrees	Longitude, decimal degrees	Depth, m	References	Entirely AMS-based	SST alignment to NGRIP	MS aligment to NGRIP	SST alignment to EDML	Combined AMS + SST alignment to NGRIP	Combined AMS + alignment to speleothems	Use of tephra chronological markers
PS2644-5	67.87	−21.77	777	Refs. ^62,63^			X				X
MD95-2010	66.68	4.57	1226	Refs. ^64,65^			X				X
RAPID-10-1P	62.98	−17.59	1237	Ref.^66^		X					X
ENAM93-21	62.74	−4.00	1020	Refs. ^67,68^			X				X
MD99-2284	62.37	−0.98	1500	Refs. ^43,69^			X				
SU90-24	62.07	−37.03	2085	Ref.^70^			X				
RAPID-17-5P	61.48	−19.54	2303	Ref.^66^		X					X
MD95-2014	60.58	−22.08	2397	Ref.^71^		X					
ODP Site 983	60.40	−23.64	1984	Refs. ^72,73^		X					
V29-202	60.38	−20.97	2658	Ref.^74^		X					
MD99-2281	60.34	−9.46	1197	Refs. ^57,58^		X					
SO82-5-2	59.17	−30.91	1416	Refs. ^75,76^		X					X
DAPC2	58.97	−9.61	1709	Ref.^77^		X					
MD95-2006	57.03	−10.06	2122	Ref.^8^		X					X
NA87-22	55.50	−14.70	2161	Refs. ^3,7,78,79^, this study		X					
GIK23415-9	53.18	−19.15	2472	Refs. ^65,80,81^		X					X
MD01-2461	51.75	−12.92	1153	Refs. ^39,65,82,83^		X					X
DSDP Site 609	49.88	−24.23	3883	Refs. ^84,85^		X					X
MD95-2002	47.45	−8.53	2174	Refs. ^86,87^		X					
MD04-2845	45.35	−5.22	4175	Refs. ^59,60^		X					
SU92-03	43.20	−10.11	3005	Ref.^88^		X					
SU90-08	43.05	−30.04	3080	Ref.^78^		X					
MD03-2697	42.16	−9.70	2164	Refs. ^89,90^		X					
MD99-2331	42.15	−9.68	2120	Refs. ^40,91,92^		X					
CH69-K09	41.76	−47.35	4100	Ref.^93^		X					
MD95-2040	40.58	−9.86	2465	Refs. ^88,94,95^					X		
MD95-2039	40.58	−10.35	3381	Refs. ^91,94^					X		
MD03-2698	38.24	−10.39	4602	Ref.^96^, this study					X		
GEOFAR-KF16	38.00	−31.13	3050	Ref.^97^	X						
MD08-3180Q	38.00	−31.13	3064	Refs. ^98,99^		X					
MD95-2041	37.83	−9.52	1123	Refs. ^40,91,100^					X		
MD95-2042	37.80	−10.17	3146	Refs. ^91,101–103^					X		
MD99-2334K	37.80	−10.17	3146	Refs. ^104,105^					X		
SU81-18	37.77	−10.18	3135	Refs. ^3,106^	X						
GEOFAR-KF13	37.58	−31.84	2690	Ref.^107^	X						
MD95-2037	37.09	−32.03	2159	Refs. ^108,109^, this study					X		
S94-2-KS04	36.87	−29.18	3400	this study	X						
KNR197-10-17GGC	36.41	−48.54	5010	Ref.^110^	X						
M39008-3	36.38	−7.07	577	Refs. ^111,112^	X						
MD08-3227G	35.27	−6.80	642	Ref.^113^, this study	X						
GIK15669-1	34.89	−7.82	2022	Ref.^114^	X						
MD04-2805Q	34.52	−7.02	859	Ref.^115^, this study	X						
OCE326-GGC5	33.70	−57.58	4550	Refs. ^116,117^	X						
KNR31-GPC5	33.69	−57.63	4583	Refs. ^7,118,119^	X						
KNR140-51GGC	32.78	−76.28	1790	Refs. ^120,121^	X						
ODP Site 1060	30.77	−74.47	3481	Refs. ^28,122^		X					
MD02-2575	29.00	−87.12	847	Refs. ^123,124^	X						
GeoB4240-2	28.89	−13.23	1358	Ref.^125^	X						
GeoB5546-2	27.54	−13.74	1172	Refs. ^126,127^	X						
OCE205-2-100GGC	26.10	−78.00	1045	Ref.^128^	X						
OCE205-2-103GGC	26.07	−78.06	965	Refs. ^129–131^	X						
GIK12392-1	25.17	−16.85	2575	Refs. ^80,132^	X						
KNR166-2-26JPC	24.33	−83.25	546	Refs. ^133–135^	X						
KNR166-2-29JPC	24.28	−83.27	648	Ref.^133^	X						
KNR166-2-31JPC	24.22	−83.30	751	Refs. ^133,136^	X						
KNR166-2-73GGC	23.74	−79.43	542	Ref.^133^	X						
GeoB7920-2	20.75	−18.58	2278	Refs. ^137,138^	X						
MD03-2705	18.08	−21.15	3085	Refs. ^139,140^	X						
CHO288-54	17.43	−77.66	1020	this study	X						
GeoB9508-5	15.50	−17.95	2384	Ref.^141^	X						
GeoB9526-5	12.43	−18.05	3233	Ref.^142^	X						
M35003-4	12.09	−61.24	1299	Ref.^143^	X						
ODP Site 1002	10.71	−65.17	892	Ref.^144^	X						
GeoB16224-1	6.65	−52.08	2510	Refs. ^145,146^	X						
EW9209-1JPC	5.90	−44.19	4056	Ref.^130^	X						
GeoB1515-1	4.24	−43.07	3129	Refs. ^147,148^	X						
CH22-KW31	3.52	5.57	1181	Ref.^149^	X						
MD03-2707	2.50	9.40	1295	Ref.^150^	X						
GeoB16206-1	−1.58	−43.02	1367	Refs. ^145,146^	X						
GeoB16202-2	−1.91	−41.59	2247	Ref.^151^	X						
MD09-3256Q	−3.55	−35.39	3537	Ref.^152^, this study						X	
GS07-150-17/1GC-A	−4.22	−37.08	1000	Refs. ^145,153^	X						
MD09-3246	−4.23	−37.10	892	this study						X	
GeoB3910-2	−4.25	−36.35	2362	Refs. ^25,154,155^						X	
ODP Site 1078C	−11.92	13.40	426	Ref.^156^	X						
GeoB1023-5	−17.16	11.01	1978	Ref.^157^	X						
GeoB3202-1	−21.62	−39.98	1090	Ref.^158^	X						
GeoB1711	−23.32	12.38	1967	Refs. ^148,159^	X						
MD08-3167	−23.32	12.38	1948	Ref.^160^, this study	X						
GL1090	−24.90	−42.50	2225	Ref.^161^	X						
ODP Site 1084	−25.51	13.03	1992	Ref.^162^	X						
GeoB6201-5	−26.67	−46.44	475	Ref.^163^	X						
KNR159-5-36GGC	−27.50	−46.50	1268	Ref.^131^	X						
KNR159-5-42JPC	−27.76	−46.63	2296	Ref.^164^	X						
GeoB1720-2	−29.00	13.84	1997	Ref.^165^	X						
MD02-2592	−32.09	14.47	2869	this study	X						
MD02-2594	−34.71	17.34	2440	Ref.^166^	X						
MD16-3511Q	−35.36	29.24	4435	this study	X						
TNO57-21	−41.10	7.80	4981	Ref.^167^				X			
MD02-2588Q	−41.33	25.83	2907	Ref.^168^	X						
MD07-3076Q	−44.15	−14.22	3770	Refs. ^4,169^				X			
JR244-GC528 *	−53.01	−58.04	598	Ref.^170^	X						

*^14^C dates calibrated using reservoir ages at 600 m water depth, based on deep sea coral data given in ref.^170^.

**Online-only Table 2:** Alignment targets.

Event	Reference record*	Age, cal. ky BP (ka)	Age, ky b2k	1 sigma, ky	Reference
Saksunavatn ash layer		10.297	10.347	0.045	Ref.^46^
end of YD	NGRIP δ^18^O	11.653	11.703	0.050	Ref.^29^
Vedde ash layer		12.121	12.171	0.057	Ref.^46^
start of YD	NGRIP δ^18^O	12.846	12.896	0.250	Ref.^31^
end of HS1 (or start of GI-1)	NGRIP δ^18^O	14.642	14.692	0.093	Ref.^29^
start of HS1	NGRIP Ca++	17.480	17.530	0.206	Ref.^33^
end of HS2 (or start of GI-2)	NGRIP δ^18^O	23.290	23.340	0.298	Ref.^29^
end of warm event within GS-3	NGRIP Ca++	24.1	24.15	0.33	Ref.^35^
start of warm event within GS-3	NGRIP Ca++	25.05	25.1	0.35	Ref.^35^
FMAZ-II ash layer		26.690	26.740	0.390	Refs. ^46,47^
start of GI-3	NGRIP δ^18^O	27.730	27.780	0.416	Ref.^29^
end of HS3 (or start of GI-4)	NGRIP δ^18^O	28.850	28.900	0.449	Ref.^29^
start of HS3	NGRIP δ^18^O	30.550	30.600	0.566	Ref.^31^
start of GI-5.1	NGRIP δ^18^O	30.790	30.840	0.566	Ref.^31^
end of GI-5.2 (or start of GS-5.2)	NGRIP δ^18^O	31.990	32.040	0.566	Ref.^31^
start of GI-5.2	NGRIP δ^18^O	32.450	32.500	0.566	Ref.^29^
end of GI-6 (or start of GS-6)	NGRIP δ^18^O	33.310	33.360	0.606	Ref.^31^
start of GI-6	NGRIP δ^18^O	33.690	33.740	0.606	Ref.^29^
end of GI-7 (or start of GS-7)	NGRIP δ^18^O	34.690	34.740	0.661	Ref.^31^
start of GI-7	NGRIP δ^18^O	35.430	35.480	0.661	Ref.^29^
end of GI-8a (start of GS-8)	NGRIP δ^18^O	36.530	36.580	0.725	Ref.^31^
start of GI-8a	NGRIP δ^18^O	36.810	36.860	0.725	Ref.^31^
end of HS4 (or start of GI-8)	NGRIP δ^18^O	38.170	38.220	0.725	Ref.^29^
start of HS4 (or start of GS-9)	NGRIP δ^18^O	39.850	39.900	0.790	Ref.^31^
start of GI-9	NGRIP δ^18^O	40.110	40.160	0.790	Ref.^29^
end of GI-10 (or start of GS-10)	NGRIP δ^18^O	40.750	40.800	0.817	Ref.^31^
start of GI-10	NGRIP δ^18^O	41.410	41.460	0.817	Ref.^29^
end of GI-11 (or start of GS-11)	NGRIP δ^18^O	42.190	42.240	0.868	Ref.^31^
start of GI-11	NGRIP δ^18^O	43.290	43.340	0.868	Ref.^29^
end of GI-12 (or start of GS-12)	NGRIP δ^18^O	44.230	44.280	0.956	Ref.^31^
end of HS5 (or start of GI-12)	NGRIP δ^18^O	46.810	46.860	0.956	Ref.^29^
start of HS5	NGRIP δ^18^O	48.290	48.340	1.000	Ref.^31^
start of GI-13	NGRIP δ^18^O	49.230	49.280	1.015	Ref.^29^
start of GI-14	NGRIP δ^18^O	54.170	54.220	1.150	Ref.^29^
NAAZ II ash layer		55.330	55.380	1.184	Ref.^46^
start of GI-15	NGRIP δ^18^O	55.750	55.800	1.196	Ref.^29^
start of GI-16	NGRIP δ^18^O	58.230	58.280	1.256	Ref.^29^
start of GI-17	NGRIP δ^18^O	59.390	59.440	1.287	Ref.^29^
beyond	NGRIP δ^18^O	beyond	beyond	1.500	guess

*NGRIP δ^18^O or air temperature versus GICC05^61,171^; NGRIP Ca++ ^61^.

## References

[1] Bard E (1994). The North Atlantic atmosphere-sea surface ^14^C gradient during the Younger Dryas climatic event. Earth and Planetary Science Letters.

[2] Sikes EL, Samson CR, Guilderson TP, Howard WR (2000). Old radiocarbon ages in the southwest Pacific Ocean during the last glacial period and deglaciation. Nature.

[3] Waelbroeck C (2001). The timing of the last deglaciation in North Atlantic climate records. Nature.

[4] Skinner LC, Fallon S, Waelbroeck C, Michel E, Barker S (2010). Ventilation of the deep Southern Ocean and deglacial CO_2_ rise. Science.

[5] Govin Aline, Michel Elisabeth, Labeyrie Laurent, Waelbroeck Claire, Dewilde Fabien, Jansen Eystein (2009). Evidence for northward expansion of Antarctic Bottom Water mass in the Southern Ocean during the last glacial inception. Paleoceanography.

[6] Vazquez Riveiros N (2010). Response of South Atlantic deep waters to deglacial warming during Terminations V and I. Earth and Planetary Science Letters.

[7] Waelbroeck C., Skinner L. C., Labeyrie L., Duplessy J.-C., Michel E., Vazquez Riveiros N., Gherardi J.-M., Dewilde F. (2011). The timing of deglacial circulation changes in the Atlantic. Paleoceanography.

[8] Austin WEN, Hibbert FD (2012). Tracing time in the ocean: a brief review of chronological constraints (60-8 kyr) on North Atlantic marine event-based stratigraphies. Quat. Sci. Rev..

[9] Björck S (1996). Synchronized Terrestrial-Atmospheric deglacial records around the North Atlantic. Science.

[10] Austin WEN, Wilson LJ, Hunt JB (2004). The age and chronostratigraphical significance of North Atlantic Ash Zone II. Journal of Quaternary Science.

[11] Liu Z (2009). Transient simulation of last deglaciation with a new mechanism for Bølling-Allerød warming. Science.

[12] Menviel L, Spence P, England M (2015). Contribution of enhanced Antarctic Bottom Water formation to Antarctic warm events and millennial-scale atmospheric CO_2_ increase. Earth and Planetary Science Letters.

[13] Pedro JB (2018). Beyond the bipolar seesaw: Toward a process understanding of interhemispheric coupling. Quaternary Science Reviews.

[14] Svensson A (2008). A 60 000 year Greenland stratigraphic ice core chronology. Climate of the Past.

[15] Lemieux-Dudon B (2010). Consistent dating for Antarctic and Greenland ice cores. Quat. Sci. Rev..

[16] Veres D (2013). The Antarctic ice core chronology (AICC2012): an optimized multi-parameter and multi-site dating approach for the last 120 thousand years. Climate of the Past.

[17] Waelbroeck C (2019). SEANOE.

[18] Lougheed, B. & Obrochta, S. MatCal: Open Source Bayesian 14 C Age Calibration in Matlab. *Journal of Open Research Software* **4** (2016).

[19] Reimer P (2013). IntCal13 and Marine13 radiocarbon age calibration curves 0–50,000 years cal BP. Radiocarbon.

[20] Hogg AG (2013). SHCal13 Southern Hemisphere calibration, 0–50,000 years cal BP. Radiocarbon.

[21] Key RM (2004). A global ocean carbon climatology: Results from Global Data Analysis Project (GLODAP). Global biogeochemical cycles.

[22] Galbraith ED, Kwon EY, Bianchi D, Hain MP, Sarmiento JL (2015). The impact of atmospheric pCO_2_ on carbon isotope ratios of the atmosphere and ocean. Global Biogeochemical Cycles.

[23] Bereiter B (2015). Revision of the EPICA Dome C CO2 record from 800 to 600 kyr before present. Geophysical Research Letters.

[24] Lougheed BC, Metcalfe B, Ninnemann US, Wacker L (2018). Moving beyond the age–depth model paradigm in deep-sea palaeoclimate archives: dual radiocarbon and stable isotope analysis on single foraminifera. Climate of the Past.

[25] Burckel P (2015). Atlantic Ocean circulation changes preceded millennial tropical South America rainfall events during the last glacial. Geophys. Res. Lett..

[26] Cheng H (2013). Climate change patterns in Amazonia and biodiversity. Nature communications.

[27] Adolphi F (2018). Connecting the Greenland ice-core and U∕Th timescales via cosmogenic radionuclides: testing the synchroneity of Dansgaard–Oeschger events. Clim. Past.

[28] Vautravers Maryline J., Shackleton Nick J., Lopez-Martinez Constancia, Grimalt Joan O. (2004). Gulf Stream variability during marine isotope stage 3. Paleoceanography.

[29] Wolff EW, Chappellaz J, Blunier T, Rasmussen SO, Svensson A (2010). Millennial-scale variability during the last glacial: The ice core record. Quaternary Science Reviews.

[30] Paillard D, Labeyrie L, Yiou P (1996). Macintosh program performs time-series analysis. EOS.

[31] Rasmussen SO (2014). A stratigraphic framework for abrupt climatic changes during the Last Glacial period based on three synchronized Greenland ice-core records: refining and extending the INTIMATE event stratigraphy. Quaternary Science Reviews.

[32] Epica, C. M (2006). One-to-one coupling of glacial climate variability in Greenland and Antarctica. Nature.

[33] Missiaen, L. *et al*. Improving North Atlantic marine core chronologies using ^230^Th-normalization. *Paleoceanography and Paleoclimatology*, 10.1029/2018pa003444 (2019). 10.1029/2018PA003444 PMC6774303 31598586

[34] Ruth, U. *et al*. Ice core evidence for a very tight link between North Atlantic and east Asian glacial climate. *Geophysical Research Letters* **34** (2007).

[35] Austin W (2012). The synchronization of palaeoclimatic events in the North Atlantic region during Greenland Stadial 3 (ca 27.5 to 23.3 kyr b2k). Quaternary Science Reviews.

[36] Bé, A. W. H. & Tolderlund, D. S. In *Micropaleontology of the oceans*, 105–149 (Cambridge University Press, 1971).

[37] Knutz PC (2002). Multidecadal ocean variability and NW European ice sheet surges during the last deglaciation. Geochemistry, Geophysics, Geosystems.

[38] Govin A (2012). Persistent influence of ice sheet melting on high northern latitude climate during the early Last Interglacial. Climate of the Past.

[39] Peck VL (2006). High resolution evidence for linkages between NW European ice sheet instability and Atlantic Meridional Overturning Circulation. Earth and Planetary Science Letters.

[40] Voelker AH, de Abreu L (2011). A review of abrupt climate change events in the Northeastern Atlantic Ocean (Iberian Margin): Latitudinal, longitudinal, and vertical gradients. Geophysical Monograph Series.

[41] Landais A., Waelbroeck C., Masson-Delmotte V. (2006). On the limits of Antarctic and marine climate records synchronization: Lag estimates during marine isotopic stages 5d and 5c. Paleoceanography.

[42] Kissel C (1999). Rapid climatic variations during marine isotopic stage 3: magnetic analysis of sediments from Nordic Seas and North Atlantic. Earth and Planetary Science Letters.

[43] Dokken TM, Nisancioglu KH, Li C, Battisti DS, Kissel C (2013). Dansgaard-Oeschger cycles: Interactions between ocean and sea ice intrinsic to the Nordic seas. Paleoceanography.

[44] Wary M (2017). Regional seesaw between the North Atlantic and Nordic Seas during the last glacial abrupt climate events. Climate of the Past.

[45] Hoff U, Rasmussen TL, Stein R, Ezat MM, Fahl K (2016). Sea ice and millennial-scale climate variability in the Nordic seas 90 kyr ago to present. Nature communications.

[46] Blockley SP (2014). Tephrochronology and the extended intimate (integration of ice-core, marine and terrestrial records) event stratigraphy 8–128 ka b2k. Quaternary Science Reviews.

[47] Abbott PM, Davies SM (2012). Volcanism and the Greenland ice-cores: the tephra record. Earth-Science Reviews.

[48] Austin WE, Abbott PM (2010). Comment: Were last glacial climate events simultaneous between Greenland and France? A quantitative comparison using non‐tuned chronologies. M. Blaauw, B. Wohlfarth, J. A. Christen, L. Ampel, D. Veres, K. Hughen, F. Preusser and A. Svensson (2009). Journal of Quaternary Science.

[49] Lougheed BC, Obrochta S (2019). A Rapid, Deterministic Age‐Depth Modeling Routine for Geological Sequences With Inherent Depth Uncertainty. Paleoceanography and Paleoclimatology.

[50] Obrochta S (2018). Mt. Fuji Holocene eruption history reconstructed from proximal lake sediments and high-density radiocarbon dating. Quaternary Science Reviews.

[51] Waelbroeck C (2019). PANGAEA.

[52] Cheng H (2018). Atmospheric 14C/12C changes during the last glacial period from Hulu Cave. Science.

[53] Schlitzer, R. Ocean Data View, http://odv.awi.de (2007).

[54] Amante, C. & Eakins, B. ETOPO 1 Arc-Minute Global Relief Model: Procedure. *Data Source and Analysis*, *National Oceanic and Atmospheric Administration NOAA* (2009).

[55] Locarnini, R. A. *et al*. World ocean atlas 2013. Volume 1, Temperature (2013).

[56] Waelbroeck C (2019). figshare.

[57] Zumaque J (2012). An ocean–ice coupled response during the last glacial: a view from a marine isotopic stage 3 record south of the Faeroe Shetland Gateway. Climate of the Past.

[58] Wary M (2015). Stratification of surface waters during the last glacial millennial climatic events: a key factor in subsurface and deep-water mass dynamics. Climate of the Past.

[59] Sanchez Goni MF (2008). Contrasting impacts of Dansgaard–Oeschger events over a western European latitudinal transect modulated by orbital parameters. Quaternary Science Reviews.

[60] Daniau A-L, Goñi MFS, Duprat J (2009). Last glacial fire regime variability in western France inferred from microcharcoal preserved in core MD04-2845, Bay of Biscay. Quaternary Research.

[61] Seierstad IK (2014). Consistently dated records from the Greenland GRIP, GISP2 and NGRIP ice cores for the past 104 ka reveal regional millennial-scale δ18O gradients with possible Heinrich event imprint. Quaternary Science Reviews.

[62] Voelker, A. H. L. Dansgaard-Oeschger events in ultra-high resolution sediment records from the Nordic Seas, PhD thesis, Kiel University, Germany (1999).

[63] Voelker AH, Haflidason H (2015). Refining the Icelandic tephrachronology of the last glacial period–the deep-sea core PS2644 record from the southern Greenland Sea. Global and Planetary Change.

[64] Dokken TM, Jansen E (1999). Rapid changes in the mechanism of ocean convection during the last glacial period. Nature.

[65] Abbott PM, Griggs AJ, Bourne AJ, Chapman MR, Davies SM (2018). Tracing marine cryptotephras in the North Atlantic during the last glacial period: Improving the North Atlantic marine tephrostratigraphic framework. Quaternary Science Reviews.

[66] Thornalley, D. J. R., Elderfield, H. & McCave, I. N. Intermediate and deep water paleoceanography of the northern North Atlantic over the past 21,000 years. *Paleoceanography* **25**, 10.1029/2009PA001833 (2010).

[67] Rasmussen TL, van Weering TCE, Labeyrie L (1996). High resolution stratigraphy of the Faeroe-Shetland channel and its relation to North Atlantic paleoceanography: the last 87 kyr. Marine Geology.

[68] Rasmussen, T. L., Thomsen, E. & van Weering, T. C. E. In *Geological processes on continental margins: Sedimentation, mass-wasting and stability* Vol. 129 (eds Stoker, M. S., Evans, D. & Cramp, A.) 255–267 (Geological Society, Special Publications, 1998).

[69] Sadatzki H (2019). Sea ice variability in the southern Norwegian Sea during glacial Dansgaard-Oeschger climate cycles. Science advances.

[70] Elliot M, Labeyrie L, Duplessy JC (2002). Changes in North-Atlantic deep-water formation associated with the Dansgaard-Oeschger temperature oscillations (60-10 ka). Quaternary Science Reviews.

[71] Manthé, S. *Variabilité de la circulation thermohaline glaciaire et interglaciaire en Atlantique Nord tracée par les foraminifères planctoniques et la microfaune benthique*, PhD thesis, University of Bordeaux 1, France (1998).

[72] Raymo M. E., Oppo D. W., Flower B. P., Hodell D. A., McManus J. F., Venz K. A., Kleiven K. F., McIntyre K. (2004). Stability of North Atlantic water masses in face of pronounced climate variability during the Pleistocene. Paleoceanography.

[73] Barker S (2015). Icebergs not the trigger for North Atlantic cold events. Nature.

[74] Oppo DW, Lehman SJ (1995). Suborbital timescale variability of North Atlantic deep water during the past 200,000 years. Paleoceanography.

[75] van Kreveld S (2000). Potential links between surging ice sheets, circulation changes and the Dansgaard-Oeschger cycles in the Irminger Sea, 60-18 kyr. Paleoceanography.

[76] Brendryen J, Haflidason H, Sejrup HP (2011). Non‐synchronous deposition of North Atlantic Ash Zone II in Greenland ice cores, and North Atlantic and Norwegian Sea sediments: an example of complex glacial‐stage tephra transport. Journal of Quaternary Science.

[77] Knutz Paul C., Zahn Rainer, Hall Ian R. (2007). Centennial-scale variability of the British Ice Sheet: Implications for climate forcing and Atlantic meridional overturning circulation during the last deglaciation. Paleoceanography.

[78] Vidal L (1997). Evidence for changes in the North Atlantic Deep Water linked to meltwater surges during the Heinrich events. Earth and Planetary Science Letters.

[79] Duplessy JC (1992). Changes in surface salinity of the North Atlantic Ocean during the last deglaciation. Nature.

[80] Vogelsang, E., Sarnthein, M. & Pflaumann, U. *δ*^18^*O Stratigraphy, chronology, and sea surface temperatures of Atlantic sediment records* (*GLAMAP-2000 Kiel*). Report 13 (Universität Kiel, Kiel, 2001).

[81] Weinelt M., Vogelsang E., Kucera M., Pflaumann U., Sarnthein M., Voelker A., Erlenkeuser H., Malmgren B. A. (2003). Variability of North Atlantic heat transfer during MIS 2. Paleoceanography.

[82] Peck V. L., Hall I. R., Zahn R., Scourse J. D. (2007). Progressive reduction in NE Atlantic intermediate water ventilation prior to Heinrich events: Response to NW European ice sheet instabilities?. Geochemistry, Geophysics, Geosystems.

[83] Peck V. L., Hall I. R., Zahn R., Elderfield H. (2008). Millennial-scale surface and subsurface paleothermometry from the northeast Atlantic, 55-8 ka BP. Paleoceanography.

[84] Broecker WS (1990). Salinity history of the northern Atlantic during the last deglaciation. Paleoceanography.

[85] Bond G (1993). Correlations between climate records from North Atlantic sediments and Greenland ice. Nature.

[86] Eynaud F., Zaragosi S., Scourse J. D., Mojtahid M., Bourillet J. F., Hall I. R., Penaud A., Locascio M., Reijonen A. (2007). Deglacial laminated facies on the NW European continental margin: The hydrographic significance of British-Irish Ice Sheet deglaciation and Fleuve Manche paleoriver discharges. Geochemistry, Geophysics, Geosystems.

[87] Toucanne S (2015). Millennial-scale fluctuations of the European Ice Sheet at the end of the last glacial, and their potential impact on global climate. Quaternary Science Reviews.

[88] Salgueiro E (2010). Temperature and productivity changes off the western Iberian margin during the last 150 ky. Quaternary Science Reviews.

[89] Naughton F (2007). Present-day and past (last 25000 years) marine pollen signal off western Iberia. Marine Micropaleontology.

[90] Naughton F (2016). Climate variability across the last deglaciation in NW Iberia and its margin. Quaternary International.

[91] Salgueiro E (2014). Past circulation along the western Iberian margin: a time slice vision from the Last Glacial to the Holocene. Quaternary Science Reviews.

[92] Naughton F (2009). Wet to dry climatic trend in north-western Iberia within Heinrich events. Earth and Planetary Science Letters.

[93] Labeyrie, L. *et al*. In *Geophysical Monograph Series 112*, *Mechanisms of global climate change at millennial time scales* (eds Clark, P., Webb, R. S. & Keigwin, L. D.) 77–98 (AGU, 1999).

[94] Schönfeld J, Zahn R, de Abreu L (2003). Surface and deep water response to rapid climate changes at the Western Iberian Margin. Global and Planetary Change.

[95] de Abreu L, Shackleton N, Schönfeld J, Hall M, Chapman M (2003). Millennial-scale oceanic climate variability of the Western Iberian margin during the last two glacial periods. Marine Geology.

[96] Lebreiro S (2009). Sediment instability on the Portuguese continental margin under abrupt glacial climate changes (last 60 kyr). Quaternary Science Reviews.

[97] Repschläger J, Garbe-Schönberg D, Weinelt M, Schneider R (2017). Holocene evolution of the North Atlantic subsurface transport. Climate of the Past.

[98] Schwab C., Kinkel H., Weinelt M., Repschläger J. (2012). Coccolithophore paleoproductivity and ecology response to deglacial and Holocene changes in the Azores Current System. Paleoceanography.

[99] Sarnthein M, Balmer S, Grootes PM, Mudelsee M (2015). Planktic and benthic 14 C reservoir ages for three ocean basins, calibrated by a suite of 14 C plateaus in the glacial-to-deglacial Suigetsu atmospheric 14 C record. Radiocarbon.

[100] Voelker A. H. L., de Abreu L., Schönfeld J., Erlenkeuser H., Abrantes F. (2009). Hydrographic conditions along the western Iberian margin during marine isotope stage 2. Geochemistry, Geophysics, Geosystems.

[101] Shackleton NJ, Hall MA, Vinent E (2000). Phase relationships between millenial-scale events 64 000–24 000 years ago. Paleoceanography.

[102] Bard E (2013). Radiocarbon calibration/comparison records based on marine sediments from the Pakistan and Iberian margins. Radiocarbon.

[103] Chabaud L, Sánchez Goñi MF, Desprat S, Rossignol L (2014). Land–sea climatic variability in the eastern North Atlantic subtropical region over the last 14,200 years: atmospheric and oceanic processes at different timescales. The Holocene.

[104] Skinner, L., Shackleton, N. & Elderfield, H. Millennial-scale variability of deep-water temperature and d18Odw indicating deep-water source variations in the Northeast Atlantic, 0–34 cal. ka BP. *Geochemistry Geophysics Geosystems* **4**, 10.1029/2003GC000585 (2003).

[105] Skinner L, Shackleton N (2004). Rapid transient changes in northeast Atlantic deep water ventilation age across Termination I. Paleoceanography.

[106] Bard E (1987). Retreat velocity of the North Atlantic polar front during the last deglaciation determined by 14C accelerator mass spectrometry. Nature.

[107] Richter, T. *Sedimentary fluxes at the mid-atlantic ridge - sediment sources, accumulation rates, and geochemical characterisation*. GEOMAR Report 73 (Christian Albrechts University in Kiel, Kiel, 1998).

[108] Labeyrie L, Waelbroeck C, Cortijo E, Michel E, Duplessy J-C (2005). Changes in deep water hydrology during the Last Deglaciation. C. R. Geoscience.

[109] Gherardi J.-M., Labeyrie L., Nave S., Francois R., McManus J. F., Cortijo E. (2009). Glacial-interglacial circulation changes inferred from231Pa/230Th sedimentary record in the North Atlantic region. Paleoceanography.

[110] Keigwin LD, Swift SA (2017). Carbon isotope evidence for a northern source of deep water in the glacial western North Atlantic. Proceedings of the National Academy of Sciences.

[111] Cacho I (2001). Variability of the western Mediterranean Sea surface temperature during the last 25,000 years and its connection with the Northern Hemisphere climatic changes. Paleoceanography.

[112] Eynaud F (2009). Position of the Polar Front along the western Iberian margin during key cold episodes of the last 45 ka. Geochemistry Geophysics Geosystems.

[113] Delivet, S. *Sedimentary expression of internal waves on Quaternary contouritic processes along the Irish and Moroccan Atlantic margins*. Ph.D. thesis, Ghent University, Belgium (2016).

[114] Sarnthein M (1994). Changes in east Atlantic deepwater circulation over the last 30 000 years: eight time slice reconstructions. Paleoceanography.

[115] Penaud A (2010). Contrasting paleoceanographic conditions off Morocco during Heinrich events (1 and 2) and the Last Glacial Maximum. Quaternary Science Reviews.

[116] McManus JF, Francois R, Gherardi J-M, Keigwin LD, Brown-Leger S (2004). Collapse and rapid resumption of Atlantic meridional circulation linked to deglacial climate changes. Nature.

[117] Carlson AE (2008). Subtropical Atlantic salinity variability and Atlantic meridional circulation during the last deglaciation. Geology.

[118] Keigwin LD, Jones GA (1994). Western North Atlantic evidence for millenial-scale changes in ocean circulation and climate. Journal of Geophysiscal Research.

[119] Keigwin LD, Jones GA, Lehman SJ (1991). Deglacial meltwater discharge, North Atlantic deep circulation and abrupt climate change. Journal of Geophysiscal Research.

[120] Keigwin Lloyd D. (2004). Radiocarbon and stable isotope constraints on Last Glacial Maximum and Younger Dryas ventilation in the western North Atlantic. Paleoceanography.

[121] Rasmussen T, Thomsen E (2012). Changes in planktic foraminiferal faunas, temperature and salinity in the Gulf Stream during the last 30,000 years: influence of meltwater via the Mississippi River. Quaternary Science Reviews.

[122] Hoogakker B, McCave I, Vautravers M (2007). Antarctic link to deep flow speed variation during Marine Isotope Stage 3 in the western North Atlantic. Earth and Planetary Science Letters.

[123] Ziegler M, Nürnberg D, Karas C, Tiedemann R, Lourens LJ (2008). Persistent summer expansion of the Atlantic Warm Pool during glacial abrupt cold events. Nature Geoscience.

[124] Nürnberg D, Ziegler M, Karas C, Tiedemann R, Schmidt MW (2008). Interacting Loop Current variability and Mississippi River discharge over the past 400 kyr. Earth and Planetary Science Letters.

[125] Henderiks J (2002). Glacial–interglacial variability of particle accumulation in the Canary Basin: a time-slice approach. Deep Sea Research Part II: Topical Studies in Oceanography.

[126] Plewa K., Meggers H., Kasten S. (2006). Barium in sediments off northwest Africa: A tracer for paleoproductivity or meltwater events?. Paleoceanography.

[127] Kuhlmann H, Freudenthal T, Helmke P, Meggers H (2004). Reconstruction of paleoceanography off NW Africa during the last 40,000 years: influence of local and regional factors on sediment accumulation. Marine Geology.

[128] Came RE, Oppo DW, McManus JF (2007). Amplitude and timing of temperature and salinity variability in the subpolar North Atlantic over the past 10 ky. Geology.

[129] Marchitto TM, Curry WB, Oppo DW (1998). Millennial-scale changes in North Atlantic circulation since the last glaciation. Nature.

[130] Curry, W. B., Marchitto, T. M., McManus, J. F., Oppo, D. W. & Laarkamp, K. L. In *Geophysical Monograph Series 112, Mechanisms of Global Climate Change at Millennial Time Scales* (eds Clark, P., Webb, R. S. & Keigwin, L. D.) 59–76 (AGU, 1999).

[131] Came, R. E., Oppo, D. W. & Curry, W. B. Atlantic Ocean circulation during the Younger Dryas: Insights from a new Cd/Ca record from the western subtropical South Atlantic. *Paleoceanography* **18**, 1086, 1010.1029/2003PA000888 (2003).

[132] Zahn R, Winn K, Sarnthein M (1986). Benthic foraminiferal δ13C and accumulation rates of organic carbon: Uvigerina peregrina group and Cibicidoides wuellerstorfi. Paleoceanography.

[133] Lynch‐Stieglitz, J., Schmidt, M. W. & Curry, W. B. Evidence from the Florida Straits for Younger Dryas ocean circulation changes. *Paleoceanography* **26**, 10.1029/2010PA002032 (2011).

[134] Lynch-Stieglitz J (2014). Muted change in Atlantic overturning circulation over some glacial-aged Heinrich events. Nature Geoscience.

[135] Valley S, Lynch‐Stieglitz J, Marchitto TM (2017). Timing of Deglacial AMOC Variability From a High‐Resolution Seawater Cadmium Reconstruction. Paleoceanography.

[136] Came Rosemarie E., Oppo Delia W., Curry William B., Lynch-Stieglitz Jean (2008). Deglacial variability in the surface return flow of the Atlantic meridional overturning circulation. Paleoceanography.

[137] Collins J (2011). Interhemispheric symmetry of the tropical African rainbelt over the past 23,000 years. Nature Geoscience.

[138] Papenfuss, T. Glazial-Interglaziale Variation von Planktonproduktivität und Nährstoffgehalten im tropisch-subtropischen Ostatlantik im Abbild der Barium-Gehalte. Report 5 (Institut für Geowissenschaften, Universität Kiel, Kiel, Germany, 1999).

[139] Jullien E (2007). Low-latitude “dusty events” vs. high-latitude “icy Heinrich events”. Quaternary Research.

[140] Skonieczny C (2019). Monsoon-driven Saharan dust variability over the past 240,000 years. Science advances.

[141] Mulitza Stefan, Prange Matthias, Stuut Jan-Berend, Zabel Matthias, von Dobeneck Tilo, Itambi Achakie C., Nizou Jean, Schulz Michael, Wefer Gerold (2008). Sahel megadroughts triggered by glacial slowdowns of Atlantic meridional overturning. Paleoceanography.

[142] Zarriess M, Mackensen A (2010). The tropical rainbelt and productivity changes off northwest Africa: A 31,000-year high-resolution record. Marine Micropaleontology.

[143] Vink A (2001). Shifts in the position of the North Equatorial Current and rapid productivity changes in the western Tropical Atlantic during the last glacial. Paleoceanography.

[144] Hughen K, Southon J, Lehman S, Bertrand C, Turnbull J (2006). Marine-derived 14 C calibration and activity record for the past 50,000 years updated from the Cariaco Basin. Quaternary Science Reviews.

[145] Voigt I (2017). Variability in mid‐depth ventilation of the western Atlantic Ocean during the last deglaciation. Paleoceanography.

[146] Zhang Y (2015). Origin of increased terrigenous supply to the NE South American continental margin during Heinrich Stadial 1 and the Younger Dryas. Earth and Planetary Science Letters.

[147] Rühlemann C (1996). Late Quaternary productivity changes in the western equatorial Atlantic: Evidence from 230Th-normalized carbonate and organic carbon accumulation rates. Marine Geology.

[148] Vidal L (1999). Link between the North and South Atlantic during the Heinrich events of the last glacial period. Climate Dynamics.

[149] Pastouret L, Chamley H, Delibrias G, Duplessy J-C, Thiede J (1978). Late Quaternary climatic changes in western Tropical Africa deduced frop deep-sea sedimentation of the Niger delta. Oceanologica Acta.

[150] Weldeab S, Lea DW, Schneider RR, Andersen N (2007). 155,000 years of West African monsoon and ocean thermal evolution. Science.

[151] Mulitza S (2017). Synchronous and proportional deglacial changes in Atlantic Meridional Overturning and northeast Brazilian precipitation. Paleoceanography.

[152] Burckel P (2016). Changes in the geometry and strength of the Atlantic meridional overturning circulation during the last glacial (20–50 ka). Climate of the Past.

[153] Freeman E (2015). An Atlantic–Pacific ventilation seesaw across the last deglaciation. Earth and Planetary Science Letters.

[154] Arz HW, Gerhardt S, Pätzold J, Röhl U (2001). Millennial-scale changes of surface- and deep-water flow in the western tropical Atlantic linked to Northern Hemisphere high-latitude climate during the Holocene. Geology.

[155] Jaeschke Andrea, Rühlemann Carsten, Arz Helge, Heil Gerrit, Lohmann Gerrit (2007). Coupling of millennial-scale changes in sea surface temperature and precipitation off northeastern Brazil with high-latitude climate shifts during the last glacial period. Paleoceanography.

[156] Kim, J. H., Schneider, R. R., Mulitza, S. & Müller, P. J. Reconstruction of SE trade‐wind intensity based on sea‐surface temperature gradients in the Southeast Atlantic over the last 25 kyr. *Geophysical Research Letters* **30**, 10.1029/2003GL017557 (2003).

[157] Kim J-H (2012). Pronounced subsurface cooling of North Atlantic waters off Northwest Africa during Dansgaard–Oeschger interstadials. Earth and Planetary Science Letters.

[158] Behling H, Arz HW, Pätzold J, Wefer G (2002). Late Quaternary vegetational and climate dynamics in southeastern Brazil, inferences from marine cores GeoB 3229-2 and GeoB 3202-1. Palaeogeography, Palaeoclimatology, Palaeoecology.

[159] Balmer S, Sarnthein M, Mudelsee M, Grootes PM (2016). Refined modeling and 14C plateau tuning reveal consistent patterns of glacial and deglacial 14C reservoir ages of surface waters in low‐latitude Atlantic. Paleoceanography.

[160] Collins J, Schefuss E, Govin A, Mulitza S, Tiedemann R (2014). Insolation and glacial–interglacial control on southwestern African hydroclimate over the past 140 000 years. Earth and Planetary Science Letters.

[161] Santos TP (2017). Prolonged warming of the Brazil Current precedes deglaciations. Earth and Planetary Science Letters.

[162] Farmer E. Christa, deMenocal Peter B., Marchitto Thomas M. (2005). Holocene and deglacial ocean temperature variability in the Benguela upwelling region: Implications for low-latitude atmospheric circulation. Paleoceanography.

[163] Portilho-Ramos R (2018). Methane release from the southern Brazilian margin during the last glacial. Scientific reports.

[164] Hoffman J. L., Lund D. C. (2012). Refining the stable isotope budget for Antarctic Bottom Water: New foraminiferal data from the abyssal southwest Atlantic. Paleoceanography.

[165] Dickson AJ (2009). Oceanic forcing of the Marine Isotope Stage 11 interglacial. Nature Geoscience.

[166] Martínez-Méndez Gema, Zahn Rainer, Hall Ian R., Peeters Frank J. C., Pena Leopoldo D., Cacho Isabel, Negre César (2010). Contrasting multiproxy reconstructions of surface ocean hydrography in the Agulhas Corridor and implications for the Agulhas Leakage during the last 345,000 years. Paleoceanography.

[167] Barker S, Diz P (2014). Timing of the descent into the last Ice Age determined by the bipolar seesaw. Paleoceanography.

[168] Ziegler M, Diz P, Hall IR, Zahn R (2013). Millennial-scale changes in atmospheric CO2 levels linked to the Southern Ocean carbon isotope gradient and dust flux. Nature Geoscience.

[169] Gottschalk J (2015). Abrupt changes in the southern extent of North Atlantic Deep Water during Dansgaard–Oeschger events. Nature Geoscience.

[170] Roberts J (2016). Evolution of South Atlantic density and chemical stratification across the last deglaciation. Proceedings of the National Academy of Sciences.

[171] Kindler P (2014). Temperature reconstruction from 10 to 120 kyr b2k from the NGRIP ice core. Climate of the Past.

