# Supplementary material for: Consistently dated Atlantic sediment cores over the last 40 thousand years
Source: Sci Data. 2019 Sep 2;6:165. doi: 10.1038/s41597-019-0173-8 (PMC6718518; doi:10.1038/s41597-019-0173-8)
Supplement: Supplementary file 2 — Supplementary figures [file 41597_2019_173_MOESM2_ESM.pdf]

Supplementary figures

Fig. S1. Example of comparison of alignment age-depth model and calibrated ^{14}C ages........ p.1

Fig. S2. Example of Nordic Seas core dated by alignment of its magnetic susceptibility record to the NGRIP ice $\delta^{18}O$ record........ p.2

Fig. S3. Example of Brazilian margin core dated by combining ^{14}C ages and alignment of its Ti/Ca record to El Condor cave $\delta^{18}O$ record.... p.3

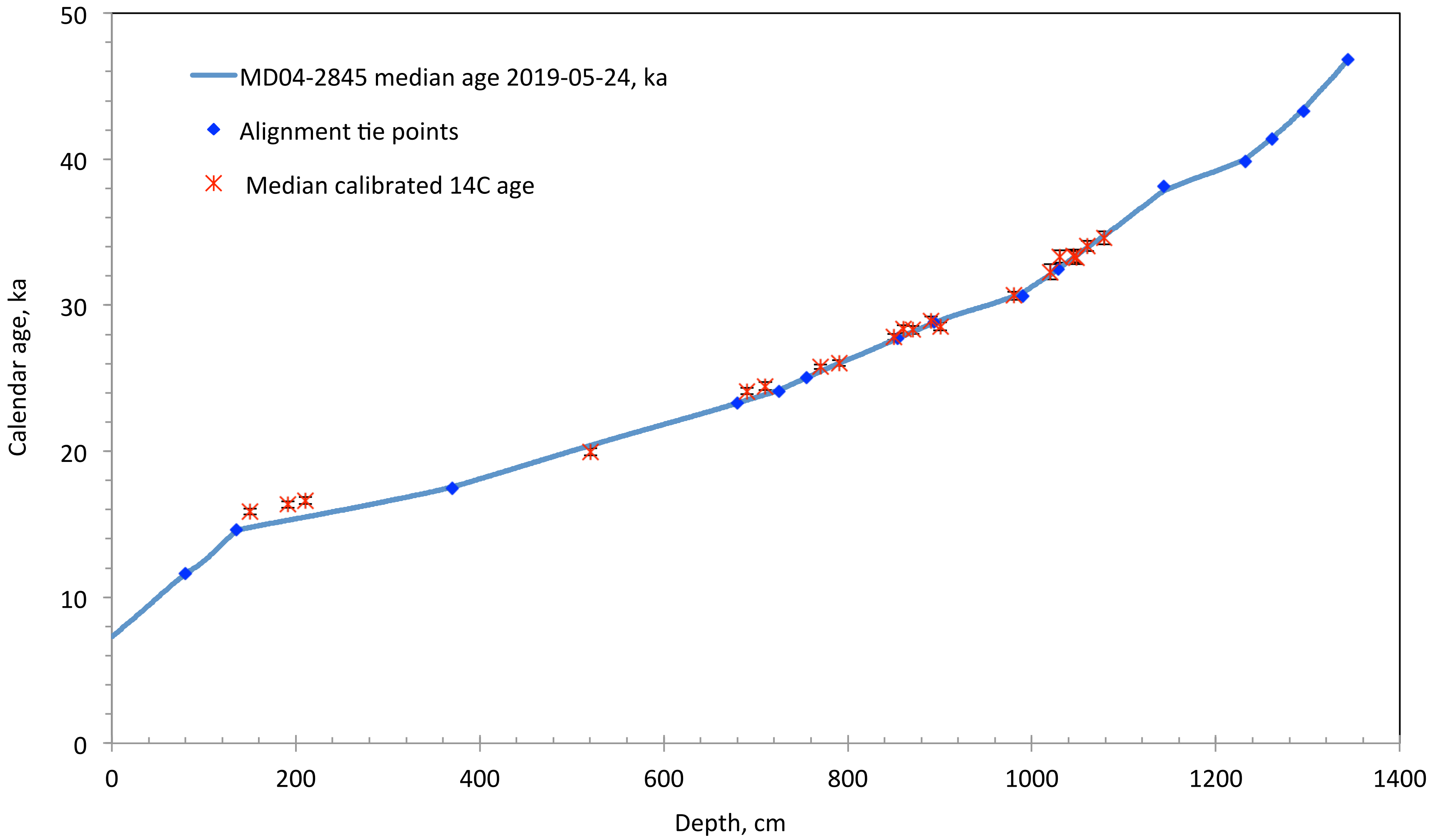

Fig. S1. Example of comparison of alignment age-depth model and calibrated ^{14}C ages. In blue: chosen alignment pointers and resulting age model computed by Undatable for core MD04-2845. Red crosses: calibrated ^{14}C ages assuming no other change to the modern surface reservoir age than the one induced by changes in atmospheric pCO_2. Modelled ages must be smaller or equal to the latter in order to avoid negative surface reservoir ages.

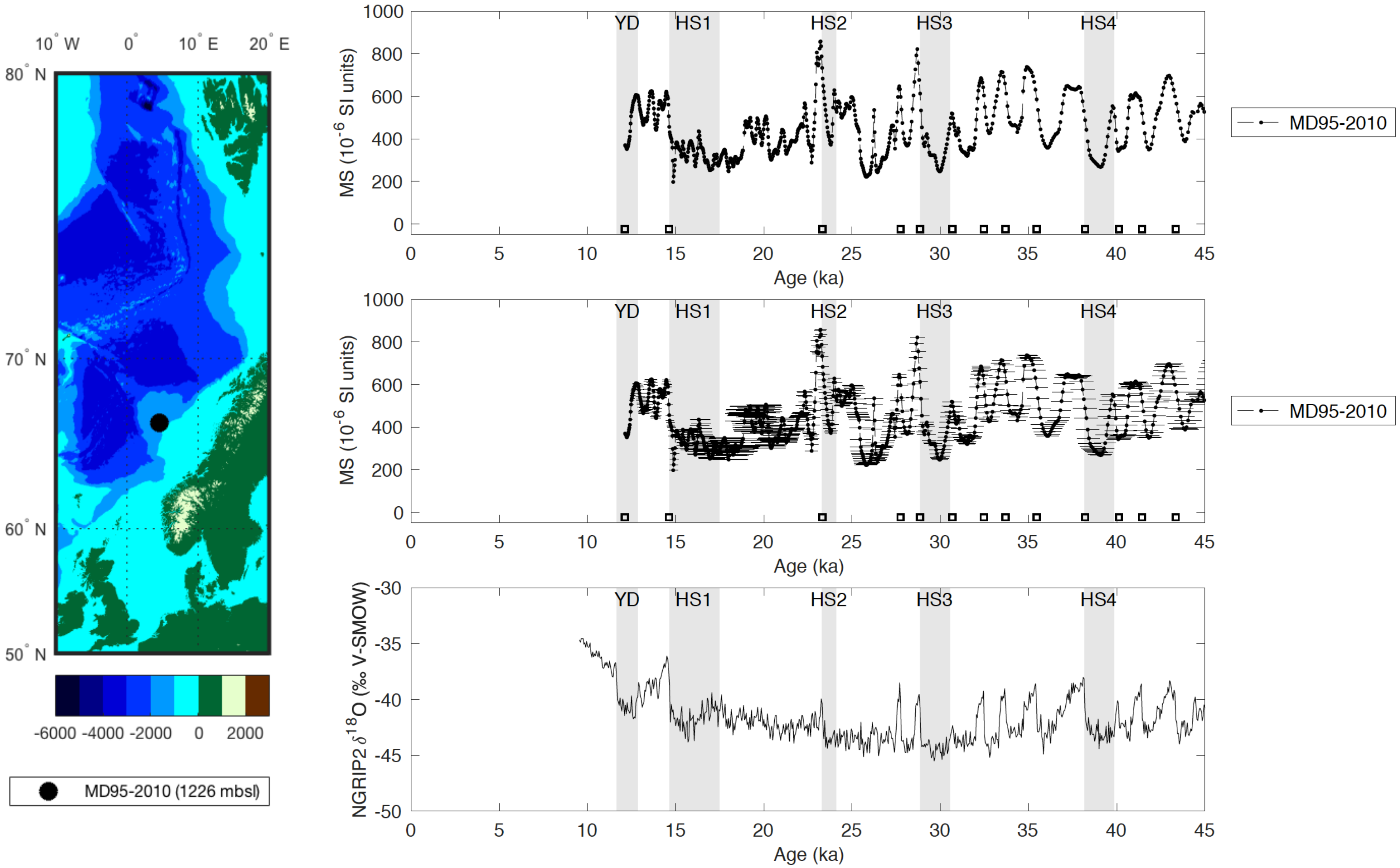

Fig. S2. Example of Nordic Seas core dated by alignment of its magnetic susceptibility record to the NGRIP ice δ^{18}O record. Top panel: magnetic susceptibility of core MD95-2010[61]. **Middle panel:** same as top panel with dating error bars (68.27% confidence intervals). **Bottom panel:** NGRIP ice δ^{18}O record on the GICC05 age scale[58]. Grey bands highlight the Younger Dryas and Heinrich stadials 1-4 chronozones as defined in Table 2.

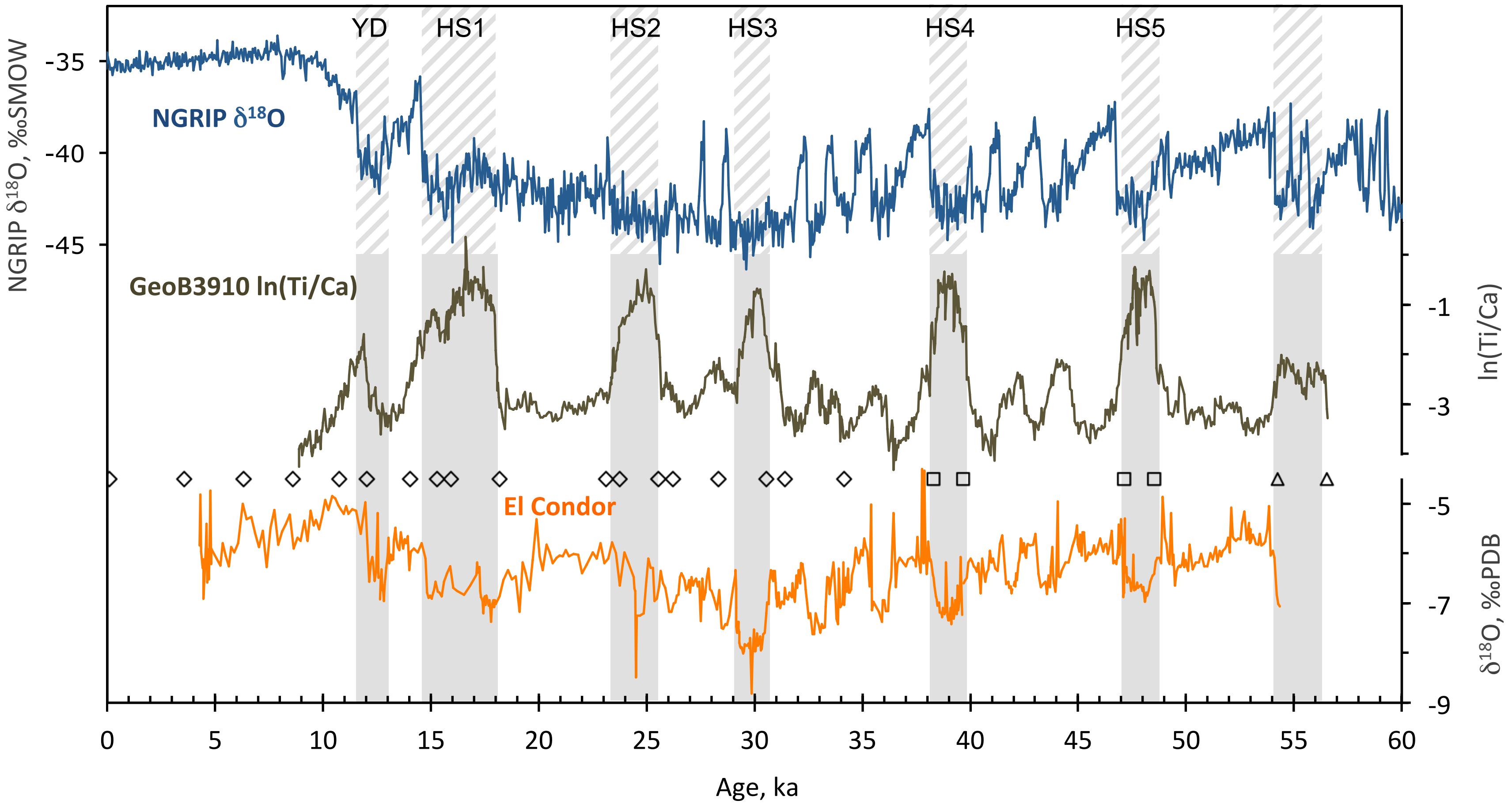

Fig. S3. Example of Brasilian margin core dated by combining ^{14}C ages and alignment of its Ti/Ca record to El Condor cave $\delta^{18}O$ record. **Upper panel:** NGRIP ice $\delta^{18}O$ record on the GICC05 age scale[58]. **Middle panel:** ln(Ti/Ca) record of core GeoB3910[152], diamonds, squares and triangles underneath indicate calibrated ^{14}C ages, alignment pointers to El Condor and NGRIP $\delta^{18}O$, respectively. **Bottom panel:** El Condor cave $\delta^{18}O$[25]. Grey bands highlight the rainfall increases recognizable in both core GeoB3910 and El Condor cave records; hatched bands highlight the Younger Dryas and Heinrich stadials 1-4 chronozones.
